# Tofu intake is inversely associated with risk of breast cancer: A meta-analysis of observational studies

**DOI:** 10.1371/journal.pone.0226745

**Published:** 2020-01-07

**Authors:** Qianghui Wang, Xingming Liu, Shengqiang Ren

**Affiliations:** Department of Urology, No. 906 Hospital of the Chinese People's Liberation Army Joint Logistic Support Force, Ningbo, China; Leibniz Institute for Prevention Research and Epidemiology BIPS, GERMANY

## Abstract

Observational studies on the association between tofu intake and breast cancer incidence have reported inconsistent results. We reviewed the current evidence and quantitatively assessed this association by conducting a dose-response meta-analysis. The electronic databases PubMed and EMBASE were searched for relevant studies published up to August, 2018. We included epidemiological studies that reported relative risks (RRs) or odds ratios (ORs) with 95% confidence intervals (CIs) for the association between tofu intake and breast cancer risk. A total of 14 studies (2 cohort studies, 12 case-control studies) were included in the meta-analysis. The overall OR of breast cancer for highest vs lowest intake of tofu was 0.78 (95% CI 0.69–0.88), with moderate heterogeneity (P = 0.011, I^2^ = 49.7%). Dose-response analysis based on 5 case-control studies revealed that each 10 g/d increase in tofu intake was associated with 10% reduction in the risk of breast cancer (95% CI 7%–13%, P = 0.037, I^2^ = 40.8%). In summary, our findings suggest an inverse dose-response association between tofu intake and risk of breast cancer. However, owing to the limitations of case-control studies, more properly designed prospective studies are warranted to confirm this association.

## Introduction

Breast cancer is the most prevalent cancer and the leading cause of cancer death in females worldwide, with an estimated 1.7 million cases and 521,900 deaths in 2012 [1]. Except for the well-known hereditary and genetic factors, the etiology of breast cancer remains largely unknown. The incidence are generally high in Western countries and low in Asia, but rapidly increase in Asian women was noted with the westernization of lifestyle, suggesting other factors such as parity, menstruation, physical activity, breast feeding, and nutrition may modify the risk of breast cancer [2]. The role of dietary factors in the cause of breast cancer has been extensively investigated, and the results have been inconsistent [3, 4]. In 2007, the World Cancer Research Fund concluded in their publication “Food, Nutrition, and the Prevention of Cancer: A Global Perspective” that the scientific data on the relation of breast cancer and dietary intake were too limited to reach a conclusion [5].

Soy foods are traditionally more relevant to Asian diets than Western diets [6, 7]. They contain high level of isoflavones, which have been shown to suppress breast cancer cells in a large number of experimental studies [8–10]. Epidemiologic studies have suggested that high intake of soy foods was associated with a reduced risk of breast cancer incidence [11, 12]. However, it is unclear which soy foods are particularly beneficial for breast cancer prevention. Tofu (bean curd), a popular food derived from soy, have long been a staple of Asian diets. The low incidence of breast cancer in Asia may be partly due to the high intake of tofu. It is now widely distributed not only to Asian countries but also to Europe and the United States, and planning tofu intervention studies is easy in areas where consumption of tofu is high, such as China and Japan. The association between tofu intake and breast cancer incidence has been considered in several studies, with conflicting results. The largest prospective study, the Japan Collaborative Cohort (JACC) Study, found no relation of breast cancer with tofu intake [13]. However, another study in Japan and several other case-control studies reported an inverse association [14–16]. To clarify the potential association between tofu intake and the risk of breast cancer, we combined all published data using a food-based meta-analytic approach.

## Methods

### Literature search and study selection

The meta-analysis was conducted in accordance with the Preferred Reporting Items for Systematic Reviews and Meta-Analyses (PRISMA) recommendation [17]. The electronic databases PubMed and EMBASE were searched for articles published up to August, 2018. We noted that there were very few studies retrieved when using “tofu” and “breast cancer” as the search terms. To broaden the search, medical subject headings (MeSH) in combination with free text searches were used, and the search terms were: (case-control OR cohort OR prospective OR retrospective) AND (tofu OR bean curd OR soy OR soyfood OR bean or diet or “soy foods” [Mesh]) AND (breast cancer or“Breast Neoplasms” [Mesh]). References cited in the included studies were also hand-searched for possible inclusions.

The identification of relevant studies was performed independently by two different authors (QW and XL), and disagreements were resolved through consultation with a third reviewer (SR). Studies were included if they fulfilled the following criteria: 1) a case–control, cohort or cross-sectional design published in the English language; 2) studies evaluating the potential association between the risk of breast cancer and dietary tofu intake; 3) provided estimators of risk, such as relative risk (RR), incidence rate ratios (IRR), hazard ratios (HR), or odds ratios (OR) with 95% confidence intervals (CIs), or reported data to calculate them. The exclusion criteria were as follows: 1) experimental study; 2) letters or case reports; 3) articles that provided inadequate data or only information for breast cancer mortality. To avoid incorporating duplicated information, multiple publications from the same author or institution were seriously scrutinized, the study with the largest number of cases was included.

### Data extraction

The study characteristics were extracted independently by two authors (QW and SR) and any disagreement was resolved by consultation with a third reviewer (XL). From each article, the following variables were recorded: first author’s name, year of publication, study design, origin of the studied population, sample size, verification of breast cancer, range of categories of tofu intake, risk estimates with corresponding 95% CIs, adjustment for potentially confounding factors (the most important potential confounders were age, age at menarche, parity, family history, and hormone replacement therapy use), and exposure assessment. Since the absolute risk of breast cancer is low, all the measures of association (such as RR, IRR and HR) are approximately equal to the estimates of OR, and the OR was used as the study outcome. When multiple ORs were available, we extracted estimates with the most comprehensive adjustment. When studies reported results separately by menopause or estrogen receptor (ER) status, both risk estimates were included separately in the analyses.

Quality assessment for each article was evaluated by according to the Newcastle Ottawa Scale (NOS), which was recommended by the Cochrane Non-Randomized Studies Methods Working Group (http://www.ohri.ca/programs/clinical_epidemiology/oxford.asp). The range of possible scores is 0–9, and we considered studies which had a total NOS score of ≥7 as high quality for the meta-analysis.

### Statistical analysis

We pooled OR for category of the highest compared with the lowest tofu intake, using the fixed or random effects model depending on the between-study heterogeneity [18, 19]. Heterogeneity was assessed by the Cochrane Q-test [19] and the Higgins *I*^*2*^ score [20]. Meta-regression analysis was used to explore the potential sources of heterogeneity. In addition, the Galbraith plot was used to detect the possible sources of heterogeneity [21]. Sensitivity analysis was performed, in which the meta-analysis estimates were computed after omission of every study in turn. Publication bias was investigated by Begg’s funnel plots [22] and the test of Egger [23]. Subgroup analyses were carried out by study design, sources of the controls (in case-control studies), number of cases, geographical region, study quality, verification of breast cancer, menopausal status, and adjustment of the most important confounders. The methods developed by Orsini [24] and Greenland and Longnecker [25] were used for the dose-response analysis. For each study, we assigned the midpoint of the tofu intake in each category as the assigned dose, and half the width of the preceding category was used to define the corresponding point for the open-ended categories. The P value for nonlinearity was calculated by testing the null hypothesis that the coefficient of the second spline is equal to zero. When a nonlinear trend was not detected, linear analysis was performed using the method described above. All statistical analyses were performed using the STATA software (version 12; StataCorp, College Station, TX, USA).

## Results

**Fig 1:**
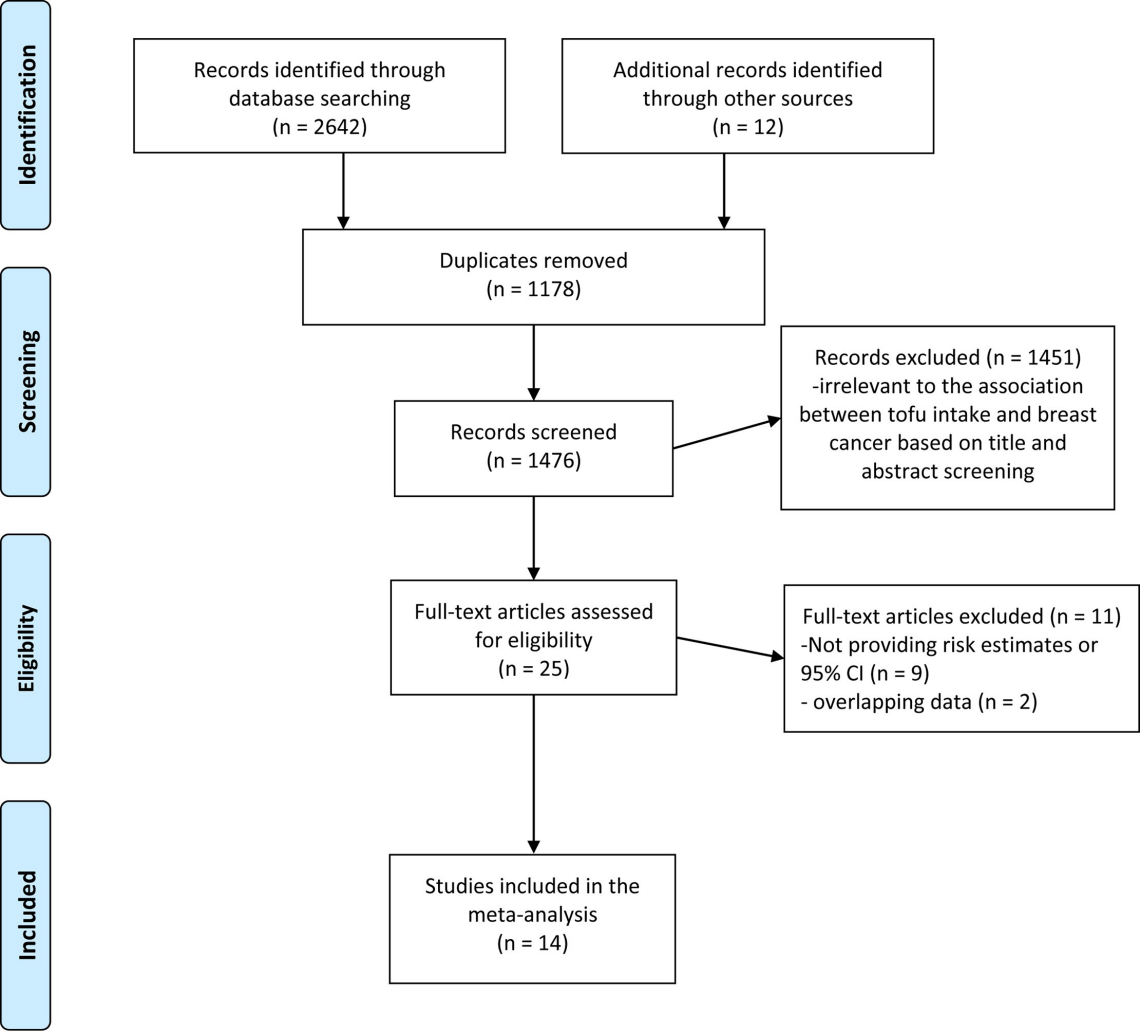
Flowchart of study selection.

Fig 1 demonstrated the detailed process of study selection from the initial search to final inclusion. A total of 2654 possible citations were identified for the overall review. After screening the titles and abstract, 2629 publications were excluded due to no relevant data, duplicates, reviews, case series, and the remaining 25 were considered as of potential values. Eleven of these 25 studies were excluded for duplicated data [26, 27] and not providing risk estimates or 95%CI [28–36]. Finally, fourteen studies, including two large-scaled cohort studies [13, 37] and twelve case–control studies [14–16, 38–46], were eligible and included in this meta-analysis. Three studies reported two separate outcomes (premenopausal and postmenopausal, estrogen receptor-positive and estrogen receptor-negative), thus there were seventeen independent outcomes included in the meta-analysis.

Among the identified articles, nine studies were conducted in Asia [13–16, 37, 39–41, 43], four in America [38, 42, 44, 45], and one in Germany [46]. Ten studies used face-to-face interview to collect information [14–16, 38–43, 45], and the remaining 4 studies used self-administered questionnaire [13, 37, 44, 46]. The diagnosis of breast cancer was based on histological findings in 9 case–control studies [14–16, 38–41, 43, 46], while the cases in 5 studies were retrieved by linkage with cancer registries [13, 37, 42, 44, 45]. The study quality scores, assessed by the Newcastle–Ottawa Quality Assessment Scale, was 6.8 for the included studies with a range of 6 to 8 points. Among these studies, ten of them achieved score 7 and one achieved score 8 [13, 14, 37–43, 46]. All these studies were considered of high quality. On the contrary, the remaining four studies were regarded as with low quality [15, 16, 44, 45]. Detailed characteristics of the included studies were presented in Table 1.

**Table 1:** Characteristics of included epidemiological studies on tofu intake and risk of breast cancer.

Authors and publication year	Study design	Country	Study period	Case/subjects	Verification of breast cancer	Exposure range	Study quality	Variables of adjustment	Exposure assessment
Wu et al. 1996 [38]	PCC	US	1983–1987	596/1554	Histologically confirmed	≥ 55 times/year vs ≤ 12 times/year	7	Age	Interview (50 items FFQ)
Witte et al. 1997 [44]	HCC	US	1957–1989	140/362	Registry	1 severvings/week vs nonconsumers	6	Age, age at menarche, parity, oral contraceptive use, alcohol consumption, body mass index, and energy.	Self-administered questionnaire (FFQ)
Key et al. 1999 [37]	Cohort	Japan	1969–1993	472/24995	Registry	≥ 5 times/week vs ≤ 1 times/week	7	Age, calendar period, city, age at time of bombing and radiation dose	Self-administered questionnaire (FFQ)
Horn-Ross et al. 2001 [42]	PCC	US	1995–1998	1326/2983	Registry	≥ 1 times/month vs nonconsumers	7	Age; race/ethnicity; age at menarche; parity; lactation; history of benign breast disease; family history of breast cancer; education; a composite variable including menopausal status, body mass index, and hormone replacement therapy use; and daily caloric intake	Interview (modified version of the Block FFQ)
Shu et al. 2001 [14]	PCC	China	1996–1998	1459/3015	Histologically confirmed	Highest vs lowest quintile	8	Intake level of rice and wheat products, age, education, family history of breast cancer, history of breast fibroadenoma, age at menarche, physical activity, ever had live birth, age at first live birth, menopausal status, and age at menopause	Interview (76 items FFQ)
Wu et al. 2002 [45]	PCC	US	1995–1998	501/1095	Registry	≥ 4 times/week vs < 1 times/month	6	Age, Asian-ethnicity, birthplace, age at menarche, pregnancy, BMI, menopausal status and use of menopausal hormones, smoking history, alcohol intake, physical activity and family history.	Interview (validated FFQ)
Hirose et al. 2003 [41]	HCC	Japan	1988–2000	1186/24349	Histologically confirmed	≥ 5 times/week vs < 1–3 times/week	7	Age, visit year, family history, age at menarche, age at menopause, parity, age at first full-term pregnancy and BMI.	Interview (FFQ)
Hirose et al. 2005 [40]	HCC	Japan	2001–2002	167/1021	Histologically confirmed	Highest vs lowest tertile	7	Age, motives for consultation, smoking, drinking, exercise, energy, family history, age at menarche, parity, age at first full-term pregnancy	Interview (119 items FFQ)
Do et al. 2007 [39]	HCC	Korea	1990–2003	359/1067	Histologically confirmed	>14.39 vs < 5.10 g/day	7	Age, education, income, age at menarche, parity, age at first live birth, history of breastfeeding, use of hormones, family history of breast cancer in a first-degree relative, frequency of exercise, physical activity, cigarette smoking, and alcohol consumption.	Interview (98 items FFQ)
Nishio et al. 2007 [13]	Cohort	Japan	1988–1997	145/20454	Registry	Almost daily < 3 times/week	7	Age, study area, family history of breast cancer, age at menopause, age at first birth, parity, use of exogenous female hormone, smoking, consumption of green leafy vegetables, walking time, body mass index, and total energy intake	Self-administered questionnaire (validated FFQ)
Suziki et al. 2008 [43]	HCC	Japan	2003–2005	678/4060	Histologically confirmed	≥ 3 times/week vs ≤ 3 times/month	7	Drinking habit, smoking habit, BMI, regular exercise, family history of breast cancer, total non-alcohol energy intake, multivitamin use, age at menarche, parity, hormone-replacement therapy, referral pattern to our hospital and age at menopause for postmenopausal women	Interview (47 items FFQ)
Kim et al. 2008 [15]	HCC	Korea	2004–2006	431/793	Histologically confirmed	Highest vs lowest quintile	6	Drinking, multivitamin use, number of children, breast feeding, and quintile of carbohydrate intake, dietary factors (quintiles of energy, vitamin E, and folate)	Interview (121 items FFQ)
Cho et al. 2010 [16]	HCC	Korea	2007–2008	424/822	Histologically confirmed	Highest vs lowest quartile	6	Age, body mass index, family history of breast cancer, current use of dietary supplements, education, occupation, smoking, alcohol intake, physical activity, menopausal status, age at menarche, parity, total energy intake and postmenopausal hormone use for postmenopausal women	Interview (validated 103 items FFQ)
Zaineddin et al. 2012 [46]	PCC	German	2001–2005	3919/11440`	Histologically Confirmed	High vs low consumption	7	Menopausal status, body mass index, education level, first-degree family history of breast cancer, history of benign breast disease, number of pregnancies, age at menarche, breastfeeding history, total number of mammograms, smoking habit, alcohol consumption, phytoestrogen supplement use, energy intake, fiber intake	Self-administered questionnaire (176 items FFQ)

Abbreviation: HCC, hospital-based case-control study; PCC, population-based case-control study; BMI, body mass index

**Fig 2:**
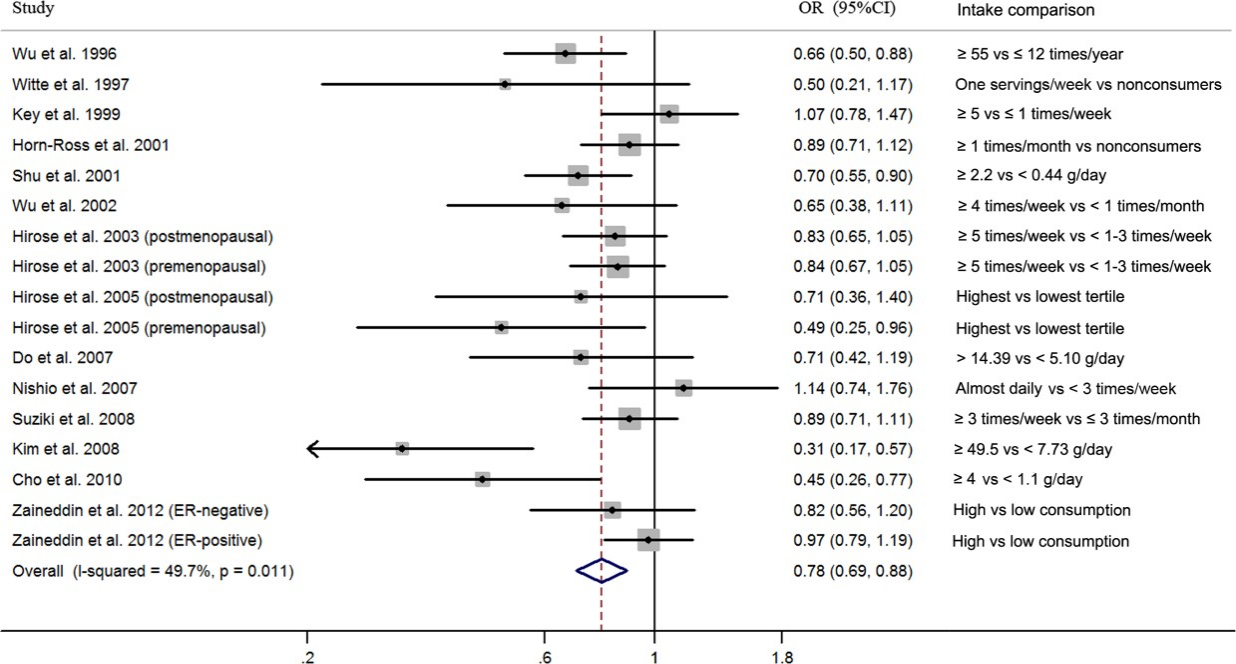
Pooled results for epidemiological studies of dietary tofu intake and risk of breast cancer.

The estimated ORs for the highest versus lowest categories of tofu intake, from each study and all studies combined, are presented in Fig 2. We found a protective effect of tofu intake on breast cancer risk (OR 0.78, 95% CI 0.69–0.88), but the heterogeneity cannot be ignored (P = 0.011 for heterogeneity, I^2^ = 49.7%). Therefore, we performed the meta-regression to explore the heterogeneity among studies (study design, number of cases, geographic area, study quality, exposure assessment, adjustment for most important confounders and breast cancer verification). As a result, only the study quality (P = 0.003) was identified as a possible source of heterogeneity. Through the Galbraith plot, we noted that 2 studies [15, 16], which had poor quality scores and were the studies with the strongest protective relationships, were the sources of heterogeneity (S1 Fig). There was no significant heterogeneity (P = 0.221, I^2^ = 20.9%) after excluding the 2 studies, and the overall association was not materially changed (OR 0.83; 95% CI, 0.76–0.91).

The sensitivity analysis was also conducted by removing one study at a time and analyzing the remaining studies (S2 Fig). The ORs ranged from 0.76 (95% CI 0.66–0.86) when the study by Zaineddin et al. [46] was excluded to 0.81 (95% CI 0.74–0.90) when the study by Kim et al. [15] was excluded suggesting that the summary OR was not dominated by any single study.

Begg’s funnel plot and Egger’s test were used to evaluate the publication bias. The shape of the funnel plots showed obvious asymmetry (S3 Fig), and the Egger’ test also showed significant publication bias (P = 0.013).

In Table 2, we pooled the OR estimates by study design (cohort and control), number of cases (> 400 and < 400), sources of the controls in case-control studies (population and hospital), and adjustment of the most important confounders (yes and no), geographical region (Western and Asian populations), study quality (high and low quality), verification of breast cancer (histologically confirmed and registry), and menopause (premenopausal and postmenopausal women). A statistically significant protective effect of tofu intake on breast cancer was observed in case-control studies (OR = 0.71; 95% CI, 0.60–0.82), while no such effect was observed in cohort studies (RR = 1.09; 95% CI, 0.81–1.38). The pool OR of ten studies showed an inverse relationship with statistical significance in premenopausal women (OR = 0.70; 95% CI, 0.52–0.87); for postmenopausal women, the pool OR of nine studies were 0.72 (95%CI 0.47–0.97), which is similar to the OR in premenopausal women. The OR estimates from other subgroup analyses showed tofu was consistently associated with reduced risk of breast cancer.

**Table 2:** Subgroup analysis of tofu intake and risk of breast cancer by study design, sources of the controls (in case-control studies), number of cases, geographical region, study quality, exposure assessment, verification of breast cancer, menopausal status and adjustment of the most important confounders.

Outcome of interest	No. of studies	OR (95% CI)	P_heterogenity_	*I*^*2*^ (%)	P for interaction
Study design					
Cohort	2	1.09 (0.85, 1.41)	0.818	0	0.125
Case-control	12	0.75 (0.66, 0.85)	0.011	49.7	
Source of control (in case-control studies)					
Population	5	0.79 (0.68, 0.90)	0.186	33.3	-
Hospital	7	0.65 (0.48, 0.81)	0.001	70.7	
Number of cases					
Larger than 400	9	0.74 (0.61, 0.87)	< 0.001	72.8	0.433
Smaller than 400	5	0.75 (0.56, 0.93)	0.195	32.1	
Geographical region					
Western countries	5	0.81 (0.69, 0.95)	0.201	31.3	0.209
Asia	9	0.76 (0.64, 0.90)	0.007	58.7	
Study quality					
High	10	0.84 (0.77, 0.92)	0.265	17.7	0.003
Low	4	0.47 (0.34, 0.64)	0.349	8.9	
Adjustment for most important confounders					
No	7	0.68 (0.53, 0.83)	0.002	67.9	0.124
Yes	7	0.81 (0.67, 0.95)	0.054	49.5	
Verification of breast cancer					
Histologically confirmed	9	0.74 (0.64, 0.85)	0.015	53.2	0.118
Registry	5	0.91 (0.72, 1.12)	0.241	27.2	
Menopause					
Premenopausal women	10	0.70 (0.52, 0.87)	< 0.001	75.5	-
Postmenopausal women	9	0.72 (0.47, 0.97)	< 0.001	84.7	

**Fig 3:**
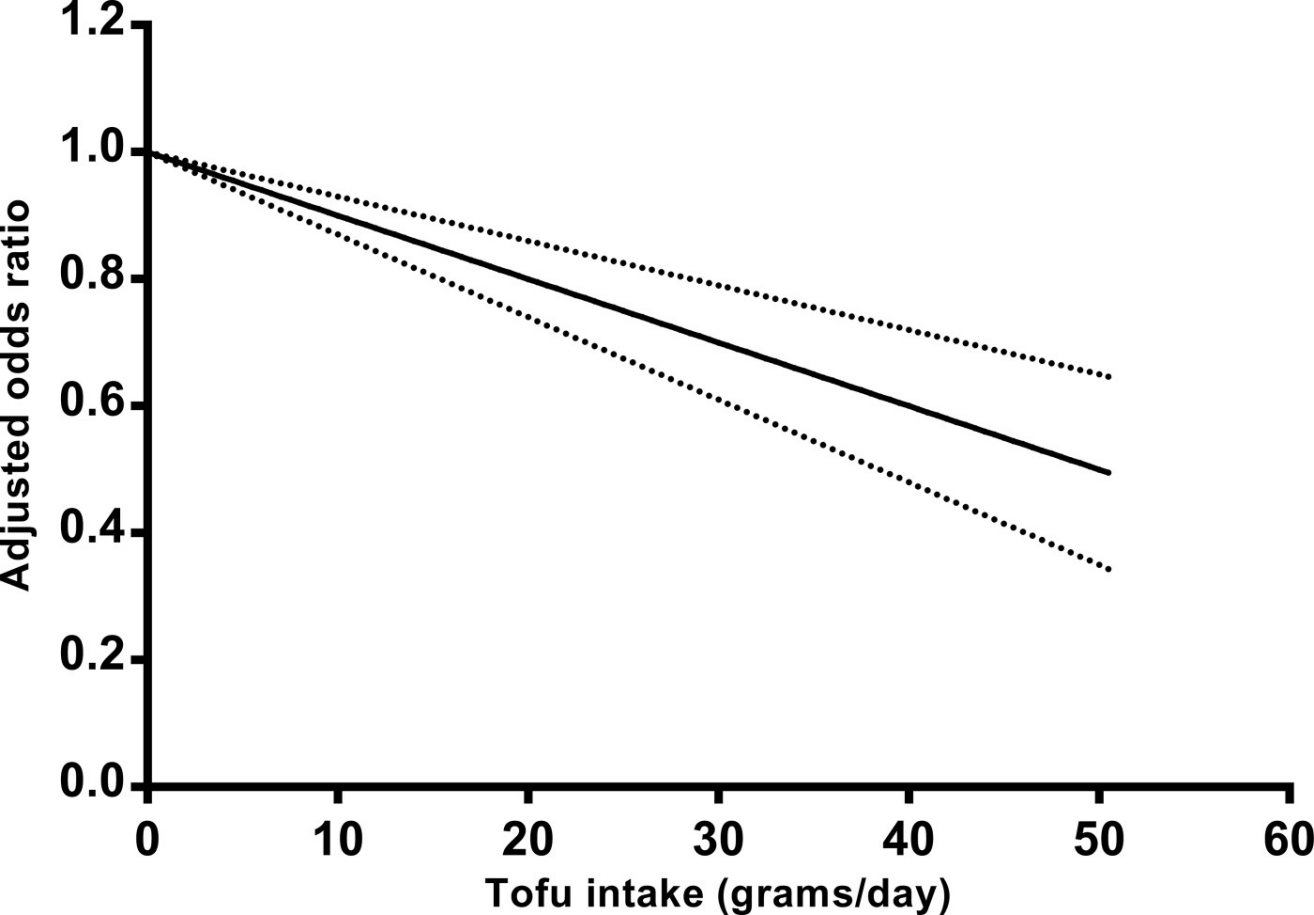
Odds ratio for breast cancer by doses of tofu intake based on the results of the dose–response meta-analyses, which included 5 studies. Solid line represents the estimated odds ratios, and the dotted lines represent the 95% confidence intervals.

Next, we assessed the dose-response relationship between tofu intake and the risk of breast cancer, which included 5 case-control studies [14–16, 39, 40] (Fig 3). The P value for the nonlinear association was 0.208, and the linear dose-response meta-analysis was used in this study. The pooled OR of breast cancer risk per 10 g/day increment in tofu intake was 0.90 (95% CI 0.87–0.93), which means there was a 10% (95% CI 7%–13%) decrease of risk of breast cancer for an increase of 10 g tofu intake per day. The result was heterogeneous (P = 0.037, I^2^ = 40.8%).

## Discussion

There is relatively little epidemiological data available for the role of tofu in the etiology of breast cancer, in part because tofu is consumed mainly in East Asian populations. In the present analysis, evidence mainly from case-control studies are supportive of a protective role of tofu intake against breast cancer. The risk of breast cancer was reduced by 22% in a comparison of the highest with the lowest category of dietary tofu intake. This finding is consistent with the results from the previous meta-analysis by Qin et al. [47] in 2006, Which investigated the relationship between tofu intake and breast cancer in a subgroup analysis. A recent meta-analysis that focused on dietary intake of soy foods and risk of breast cancer failed to detect a significant association between tofu intake and the risk of developing breast cancer [48]. However, only two prospective studies were included in the tofu analysis. We included 6 studies published after 2006 that were not included in the previous meta-analysis by Qin et al. [47] and explored the dose-response relationship between tofu and breast cancer risk. The results indicated that intake of tofu was associated with a significant 10% reduction in risk for every 10 g/d increment of dietary tofu intake.

Heterogeneity was detected in this meta-analysis in overall analysis. The Galbraith plot suggested that the observed heterogeneity seemed to be explained by Cho et al’s [16] and Kim et al’s [15] studies, which had low quality scores (both were 6) and were the studies with the strongest protective relationships. After excluding these two studies, the association between tofu intake and the risk of breast cancer was not significant changed (OR 0.83; 95% CI, 0.76–0.91; P = 0.221, I^2^ = 20.9%). In addition, the meta-regression analysis showed that the quality of the studies was a possible source of heterogeneity. When we stratified by study quality in the subgroup analyses, the association was weaker for studies of high quality (OR 0.84, 95% CI 0.77–0.92) than low quality (OR 0.47, 95% CI 0.34–0.64), with no evidence of study heterogeneity (P = 0.265, I^2^ = 17.7%; P = 0.349, I^2^ = 8.9%, respectively). These results suggested that several low-quality studies may have led to a lower risk estimate, although the combined ORs were still significant in the subgroup analyses for high-quality studies.

Tofu is a versatile ingredient with many health benefits. As a particular soy food, It also contains high concentration of isoflavones, which possess both estrogen-dependent and -independent properties that potentially inhibit the development of breast cancer [49]. It has been hypothesized that isoflavones protect against breast cancer through competitive binding to estrogen receptors. Genistein, which is the simplest isoflavonoid, is an effective inhibitor of cancer cell growth in various breast cancer cell lines, probably via the inhibition of tyrosine protein kinases and other enzymes that are associated with signal transduction of cellular growth factors [50, 51]. Published meta-analyses [11, 12, 47] have shown that soy isoflavones intake could reduce the risk of breast cancer. However, associations were only significant in high soy-consuming Asian populations, and no significant associations were found in low soy-consuming Western populations [12]. In the present analysis, the association between tofu intake and breast cancer risk was both statistically significant among women in Asian (OR 0.76, 95% CI 0.64–0.90) and Western populations (OR 0.81, 95% CI 0.69–0.95), indicating that other bioactive components in tofu might be associated with chemoprevention. For example, tofu can have a high calcium content depending on the coagulants used in manufacturing (e.g. calcium chloride, calcium sulfate), and calcium may serve as a potential regulator in breast cancer cell proliferation [52]. A meta-analysis of eleven prospective cohort studies suggest an inverse dose-response association between calcium intake and breast cancer risk [53].

Some limitations in our meta-analysis should be acknowledged. First, despite the strong inverse association between tofu intake and risk of breast cancer, our finding was based on two cohort studies and a large number of case-control studies, suggesting that our conclusion depend mainly on the case-control studies. Relatively speaking, case-control studies inevitably suffer some drawbacks like recall and selection biases, and thus our results should be interpreted with caution. Second, as a meta-analysis of observational studies, the potential impacts of unknown confounding factors on our findings cannot be completely excluded. For example, high tofu intake is characteristic of diets high in fruits and vegetables, low fat intake and increased physical activity, which are associated with lower breast cancer risk, although individual studies have considered a wide range of potential confounders in their analyses except for the study by Wu et al. [38], which have adjusted only for age. Third, we find a publication bias both visually and in formal statistical testing. Although we used loose search criteria, only published studies in English were included for this meta-analysis, and we did not attempt to gain access to studies in other languages and unpublished results. Small negative studies are less likely to be published, and grey literature, due to its diverse origins and unpublished nature, may also be difficult to find. Fourth, ER/progesterone receptor (PR) status is the important indicator for predicting efficacy of endocrine therapy and prognosis in breast cancer. Since soy isoflavones reduce breast cancer risk probably by binding to ERs, the benefit of tofu intake might be more pronounced among women with ER-positive breast cancer. However, the present meta-analysis was unable to assess risks by hormone receptor status because only one study provided stratified analysis for ER, which showed that soybean product intake was associated with a significantly decreased risk of ER-positive breast cancer, with odds ratios in the top tertile of intake of 0.74 (95% CI, 0.58–0.94), but the result were not significant in ER-negative breast cancer [43]. Fifth, there are more advanced tools available for quality assessment of studies cross-sectional and cohort studies, such as quality of cohort studies (Q-Coh) and risk of bias in nonrandomized studies of interventions (ROBINS-I), however, we only used the NOS to rate the individual articles, which might underestimate the risk of bias of studies [54, 55]. Sixth, tofu is very versatile as a food; it can be further processed into various secondary tofu products, including deep-fried tofu, grilled tofu, frozen tofu, dried-frozen tofu, fermented tofu, which may have different effects on breast cancer. However, tofu intake was generally not the main focus of the included studies, and results for specific types of tofu were not available except for one study by Hirose et al,[40] preventing us from distinguish the different types of tofu in the meta-analysis. Seventh, quintile-based comparisons are more appropriate for the dose-response analysis. However, among the five studies included in the dose-response analysis, two were quintile-based comparisons, one quartile-based, one tertile-based, and one used g/day for each category point. This heterogeneity should be taken into account when interpreting the dose-response results. Finally, this meta-analysis was not submitted to any systematic review register, which might decrease the credibility of the study, although we followed the Preferred Reporting Items for Systematic Reviews and Meta-Analyses (PRISMA) recommendation.

In conclusion, evidence from case-control studies suggested an inverse dose-response association between tofu intake and breast cancer risk. Further well-designed prospective studies focusing on this association are necessary to confirm our findings.

## Supporting information

S1 PRISMA Checklist Preferred Reporting Items for Meta-Analyses (PRISMA) statement checklist. (DOC)

S1 Fig Galbraith plot showing that 2 studies might contribute to heterogeneity. (TIF)

S2 Fig Sensitivity analysis was performed by removing each study in turn and recalculating the pooled risk estimates. (TIF)

S3 Fig Publication bias estimated by Begg’s funnel plot. (TIF)
