# Supplementary figures and images for: Tofu intake is inversely associated with risk of breast cancer: A meta-analysis of observational studies
Source: PLoS One. 2020 Jan 7;15(1):e0226745. doi: 10.1371/journal.pone.0226745 (PMC6946133; doi:10.1371/journal.pone.0226745)

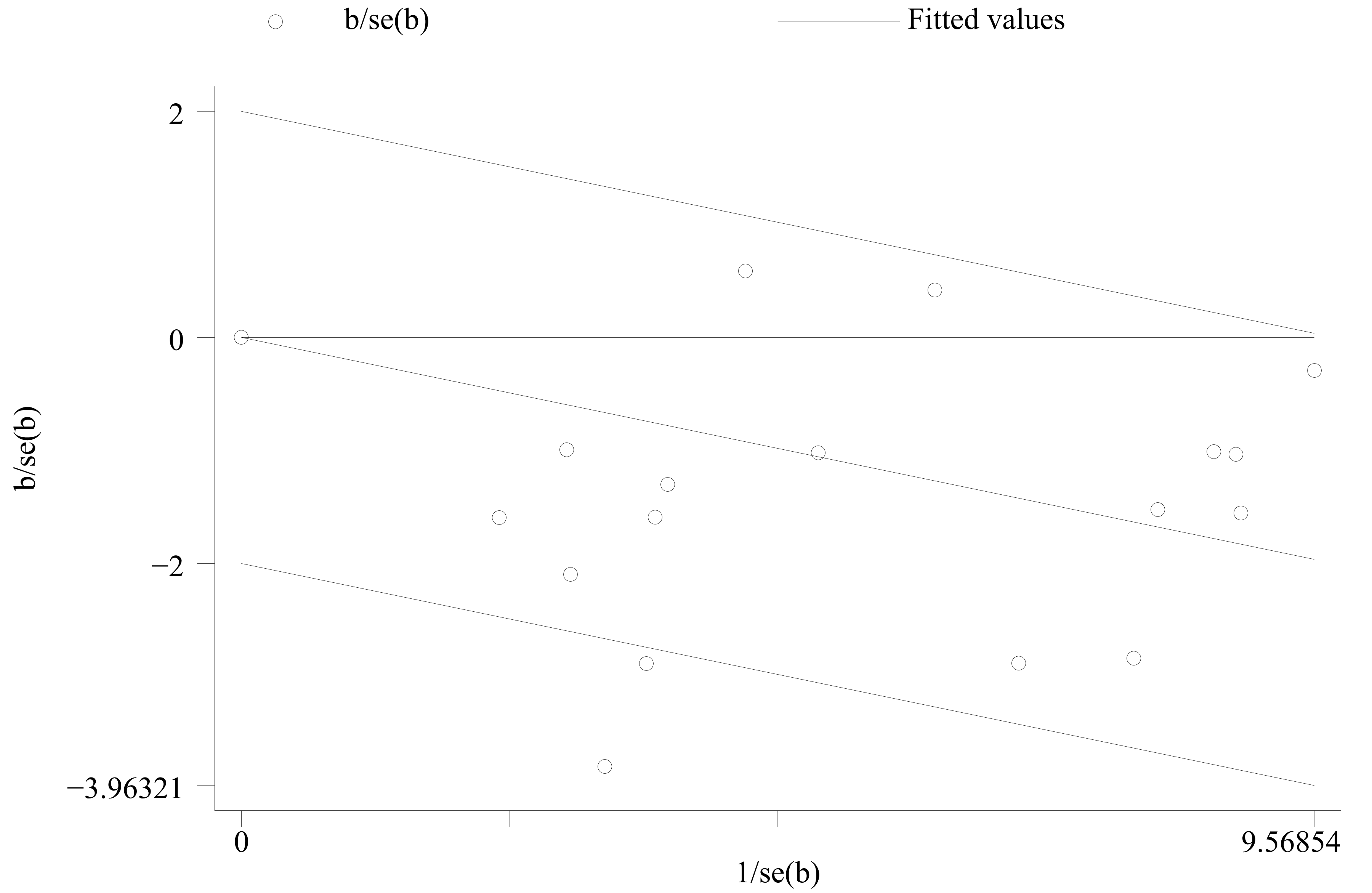

Supplement: S1 Fig — (TIF) [file pone.0226745.s002.tif]

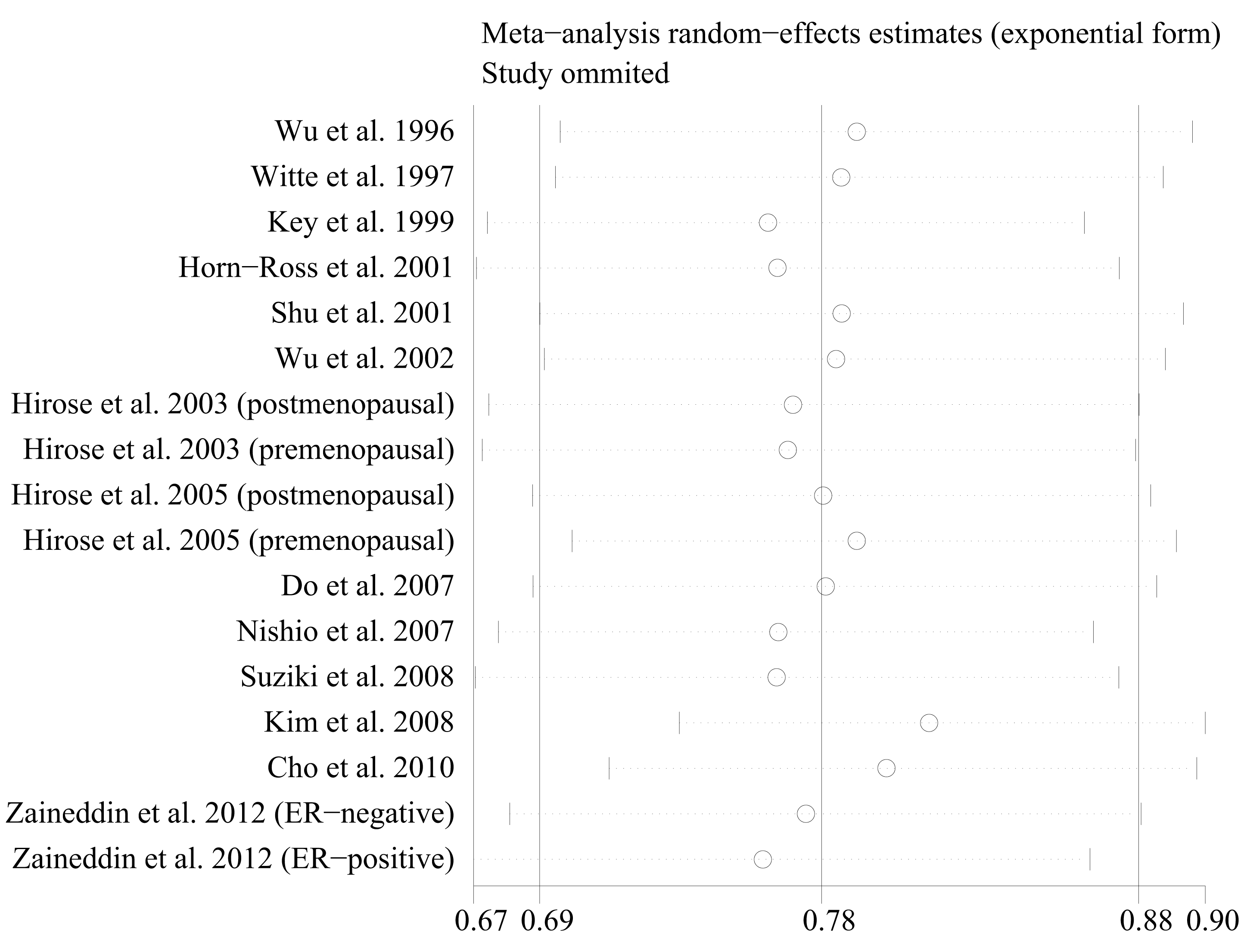

Supplement: S2 Fig — (TIF) [file pone.0226745.s003.tif]

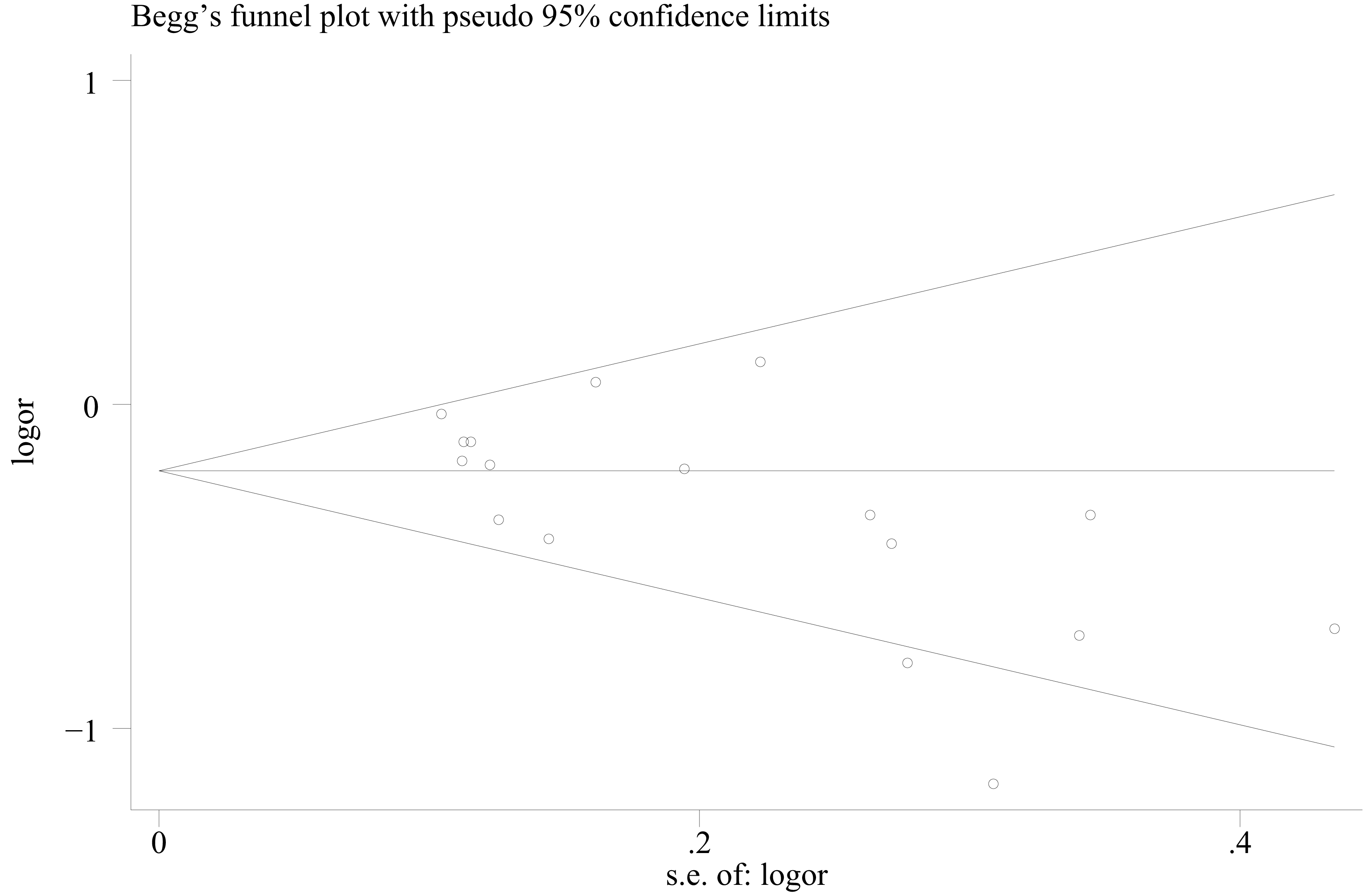

Supplement: S3 Fig — (TIF) [file pone.0226745.s004.tif]
